# Daurisoline attenuates H_2_O_2_-induced chondrocyte autophagy by activating the PI3K/Akt/mTOR signaling pathway

**DOI:** 10.1186/s13018-023-03717-5

**Published:** 2023-03-27

**Authors:** Yang Zhang, Wenguang Liu, Zhonghao Liu, Yi Liu

**Affiliations:** Department of Orthopaedics, The Second Hospital, Cheeloo College of Medicine, Shandong University, 247 Beiyuan Street, Jinan, 250033 Shandong People’s Republic of China

**Keywords:** Osteoarthritis, Daurisoline, Autophagy, Apoptosis, PI3K/AKT/mTOR signaling pathway

## Abstract

**Background:**

Osteoarthritis (OA) is a chronic degenerative joint disease characterized by cartilage degeneration and intra-articular inflammation. Daurisoline (DAS) is an isoquinoline alkaloid isolated from Rhizoma Menispermi, whose antitumor and anti-inflammatory pharmacological effects have been demonstrated, but the effects of DAS on OA have rarely been researched. In this study, we aimed to explore the potential role of DAS in OA and its partial mechanism.

**Materials and methods:**

The cytotoxicity of H_2_O_2_ and DAS toward chondrocytes was detected by the Cell Counting Kit-8 assay. Safranin O staining was used to detect chondrocyte phenotype changes. Cell apoptosis was measured by both flow cytometry and quantitative analysis of the protein levels of the apoptosis-related factors Bax, Bcl-2 and cleaved caspase-3 by western blot. Western blotting and immunofluorescence were used to assess the expression of the autophagy-related proteins LC3, Beclin-1 and p62. In addition, key signal pathway targets and matrix-degrading indicators were measured by western blot.

**Results:**

Our results indicated that H_2_O_2_ induced human chondrocyte apoptosis and activated autophagy in a dose-dependent manner. DAS treatment dose-dependently reversed the expression of apoptosis-related proteins (Bax, Bcl-2 and cleaved caspase3) and the apoptosis rate induced by H_2_O_2_. Western blot and immunofluorescence analyses showed that DAS decreased the H_2_O_2_-induced upregulation of the autophagy marker Beclin-1 and the LC3 II/LC3 I ratio and upregulated the p62 protein level. Mechanistically, DAS inhibited autophagy through the activation of the classical PI3K/AKT/mTOR signaling pathway and protected chondrocytes from apoptosis. In addition, DAS alleviated the H_2_O_2_-induced degradation of type II collagen and the high expression of matrix metalloproteinase 3 (MMP3) and MMP13.

**Conclusion:**

Our research demonstrated that DAS alleviated chondrocyte autophagy caused by H_2_O_2_ through activation of the PI3K/AKT/mTOR signaling pathway and protected chondrocytes from apoptosis and matrix degradation. In conclusion, these findings suggest that DAS may serve as a promising therapeutic strategy for OA.

## Introduction

With the acceleration of the global population aging trend, OA has become nearly universal among the middle and elderly worldwide [1, 2]. As a type of chronic degenerative joint disease, OA is characterized by progressive articular cartilage degeneration associated with multiple factors, such as metabolic disorders of the cartilage matrix, chondrocyte apoptosis, subchondral bone remodeling, and intra-articular inflammation [3–5]. The aggravation of the disease leads to total cartilage loss and destruction and ultimately causes limited movement, joint dysfunction, and even disability [6, 7]. Although some medications and physical therapy can alleviate OA symptoms and slow disease progression by improving cartilage metabolism and repairing cartilage, no effective treatment can reverse the progression of the disease [8, 9]. Therefore, it is imperative to research and develop more effective treatments.

Autophagy, which is closely related to apoptosis, is an intracellular metabolic pathway of self-degradation that is highly conserved. Autophagy provides extra energy for cells to combat antagonistic conditions, maintain intracellular homeostasis, and regulate the physiological function of cells by eliminating abnormal organelles and unnecessary proteins [10–12]. Autophagy is activated under oxidative stress, but excessive oxidative stress that exceeds the tolerance of cells will cause dysfunctional autophagy [13]. Unrestrained autophagy may lead to autophagic injury and cell apoptosis and even aggravate disease progression [14–16].

Autophagy is involved in physiological processes such as apoptosis regulation and energy metabolism in chondrocytes. In the initial phase of OA, autophagy acts as an adaptable reaction to prevent cell damage. However, excessive autophagy can be activated along with apoptosis as a substitute for cell death in the later period of OA [17]. Autophagic cell death promotes chondrocyte apoptosis and further exacerbates cartilage degeneration. Therefore, chondrocyte therapy based on the regulation of autophagy might be an essential strategy for OA treatment.

Plant-derived traditional Chinese medicine has attracted increasing attention by virtue of its efficacy and safety [18, 19]. Daurisoline (DAS) is an isoquinoline extracted from the Chinese herbal medicine Rhizoma Menispermi. Earlier research has shown the potential pharmacological effects of DAS for treating certain diseases, such as its modulation of focal ischemia/reperfusion injury, platelet aggregation, and arrhythmia [20, 21]. Furthermore, DAS has potential autophagy inhibition and exhibits anti-inflammatory and antitumor activity [22–24]. The potential bioactivity of DAS on OA, especially for autophagy, and its underlying mechanisms remain uncertain. This study established a chondrocyte damage model by treating chondrocytes with H_2_O_2_, aiming to explore the role of DAS in antioxidative autophagy and elucidate some of its mechanism of action. Furthermore, the effect of DAS on articular cartilage degeneration was investigated for the first time in vitro and in vivo, hoping to provide a new therapeutic strategy for OA.

## Materials and methods

### Patients and ethics statement

In this study, 20 patients with OA who underwent knee arthroplasty surgery at the Second Hospital of Shandong University from May 2020 to June 2021 were selected for primary human chondrocyte extraction. OA was diagnosed according to the American College of Rheumatology (ACR) knee OA criteria, and it was classified based on the Kellgren-Lawrence scoring system. The number of patients with each grade was 12 with grade 2 and 8 with grade 3. Our study was approved by the Ethics Committee of the Second Hospital of Shandong University (approval number: KYLL-2020LKJOA-0026). In addition, all patients signed the informed consent form.

### Primary human chondrocyte extraction and culture

Chondrocytes were isolated from cartilage tissue samples taken from all 20 patients with OA included in the study. We only selected macroscopic healthy specimens around the joint wear surface for cell extraction [25, 26]. Briefly, the cartilage tissues were washed with PBS 3 times to remove residual blood. Next, we cut the cartilage tissue into pieces of approximately 1 mm^3^ with ophthalmic scissors, placed they into a centrifuge tube, and added PBS to wash they three times. The cartilage tissue was digested with 0.2% type II collagenase overnight at 37 °C. The cells were separated from the digested cartilage tissue with a 200-mesh filter. After centrifugation of the cell suspension with the supernatant removed, chondrocytes were seeded in DMEM/F12 medium containing 10% fetal bovine serum (HyClone, USA) and incubated at 37 °C in an incubator with 5% CO_2_. After a 3-day incubation, the cell growth status was investigated, and the medium was replaced. The culture medium was changed every 3 days. The cells were passaged when they had grown to 80% to 90% confluency. The chondrocytes were used in experiments after at least three passages.

### Cell viability analysis

The cytotoxicity of H_2_O_2_ and DAS on chondrocytes was detected by the Cell Counting Kit-8 (CCK-8; Beyotime, Beijing, China) assay. Primary human chondrocytes (3000 cells per well) were seeded in a 96-well plate for 24 h for preincubation. First, H_2_O_2_ (Aladdin, Shanghai, China) and DAS (Yuanye Bio-technology Co, Shanghai, China, cat. no. B20095) were used to stimulate cells, and then 10 μl of CCK-8 dye was added to each well, followed by incubation in a 96-well plate at 37 °C protected from light for 2–3 h. The absorbance value (OD value) of each well at a wavelength of 450 nm was calculated by a microplate reader (Bio-Rad, USA).

### Safranin O staining

Safranin O staining was used to detect chondrocyte phenotype changes. Briefly, chondrocytes were seeded in 6-well plates, prestimulated with H_2_O_2_ (200 μM) for 4 h, and then treated with DAS (2.5 and 5 μM) for 24 h. Next, the cells were fixed with 4% paraformaldehyde at room temperature for 30 min. After being washed with PBS three times, the cells were stained with safranin O solution (Solarbio, Beijing, China) at a concentration of 0.1% for 5–10 min. Finally, the cells were washed with PBS three times and then imaged by light microscopy (Leica, Germany, magnification ×100). The statistical processing was done with ImageJ, and values were normalized to the control group.

### Chondrocyte treatment

Chondrocytes were seeded in 96-well or 6-well plates and treated with different concentrations of H_2_O_2_ (0, 10, 50, 100, 200, 300, 400 and 500 μM) and DAS (0, 1, 2.5, 5, 10, 15 and 20 μM) for 4 h and 24 h to detect cell viability and chondrocyte phenotypic changes. Based on these results, for experiments the cells were pretreated with DAS (2.5 and 5 μM) for 24 h and stimulated with H_2_O_2_ (200 μM) for 4 h. In addition, cells were collected for a western blot assay to determine the levels of autophagy markers and apoptosis-related factors, and immunofluorescence was performed to test Beclin-1. To detect the involved signal pathways, we divided the cells into 4 groups that underwent different treatments: control group, H_2_O_2_, H_2_O_2_ + DAS and H_2_O_2_ + IGF-1 as a PI3K/Akt/mTOR pathway activator to stimulate chondrocytes. We detected changes in related factors by western blot.

### Protein extraction and western blot

After appropriate treatment, cells were lysed for total protein extraction by RIPA Lysis Buffer (Beyotime) containing protease and phosphatase inhibitors, and the protein concentration was measured using a BCA assay kit (Beyotime, Beijing, China). Then, 20 μg of whole proteins from each group was loaded into 10% or 12% sodium dodecyl sulfate‒polyacrylamide gel electrophoresis (SDS-PAGE) gels for separation and transferred to polyvinylidene difluoride (PVDF) membranes. The membranes were blocked with TBST supplemented with 5% nonfat dried milk and incubated with primary antibodies at 4 °C overnight. Primary antibodies against LC3B (1:1000, CST), p62 (1:2000, Abcam), Beclin-1 (1:1000, CST), BAX (1:1000, Abcam), Bcl-2 (1:2000, Abcam), caspase-3 (1:1000, CST), PI3K (1:500, CST), p-PI3K (1:500, CST), AKT (1:500, CST), p-AKT (1:500, CST), mTOR (1:500, CST), p-mTOR (1:500, CST), Collagen II (1:500, Abcam), MMP-3 (1:1000, CST) and MMP-13 (1:1000, CST) were used in this study. After washing the membranes three times, the membranes were incubated with the relevant secondary antibodies for 1 h at room temperature. The protein bands were evaluated by an enhanced ECL kit (Millipore, USA) and a Chemiluminescence Imaging System (Tanon-4800, Shanghai, China), and the data were analyzed by ImageJ software (National Institutes of Health, USA).

### Flow cytometry

Apoptosis of chondrocytes was evaluated by an annexin V-fluorescein isothiocyanate (FITC)/PI kit (Bestbio, China). Briefly, chondrocytes were seeded into 6-well plates and treated for 6 h with serum-free DMEM to ensure synchronization. Next, chondrocytes were treated accordingly with the indicated concentrations of H_2_O_2_ and DAS. After treating the cells with 0.25% trypsin, the cells were collected, rinsed twice with PBS, centrifuged, and resuspended in 100 μl binding buffer. The cell suspension was then thoroughly mixed with 5 μl of annexin V-FITC staining solution and 5 μl propidium iodide (PI) and kept in the dark at room temperature for 15 min, followed by the addition of 400 μl of 1× binding buffer. Cell apoptosis was detected by flow cytometry (BD Accuri™ C6 Plus) at a wavelength of 488 nm. The data were analyzed using FlowJo V10.8.1 software (Tree Star).

### Immunofluorescence

Chondrocytes were seeded into 20-mm confocal Petri dishes and starved with serum-free DMEM for 24 h after their confluency reached 80%. We then randomly divided chondrocytes into H_2_O_2_, H_2_O_2_ + DAS, and control groups. After washing three times with PBS, the cells were fixed for 15 min with 4% paraformaldehyde and then permeabilized with PBS containing 0.3% Triton X-100 for 15 min, followed by a PBS wash. At room temperature, the cells were blocked with 5% BSA for 1 h. Afterward, the samples were incubated with the rabbit anti-human Beclin-1 antibody (Abcam, Britain; 1:200) at 4 °C overnight. Next, the samples were washed with PBS 3 times and incubated with fluorescein isothiocyanate-conjugated secondary antibody for 1 h at room temperature in the dark. Finally, we stained the cell nuclei with DAPI in darkness for 5 min and sealed the slides with an anti-fluorescent quenching agent. Immunofluorescence analysis was performed with a Leica DMi8 fluorescence microscope. The intensity of fluorescence was measured using ImageJ and normalized to the DAPI signal.

### Statistical analysis

Experimental data were analyzed using GraphPad Prism 8.0 (GraphPad Software Inc., San Diego, California, USA), and the measurements are expressed as mean ± standard deviation (SD). The two groups were compared using Student’s t test. One-way ANOVA was used for intergroup comparisons of > 3 groups. At least three independent replicate experiments were done to obtain all data. Differences were statistically significant at P < 0.05 and are marked with *P < 0.05, **P < 0.01, and ***P < 0.001.

## Results

### H_2_O_2_ activates apoptosis and autophagy in human chondrocytes

**Fig. 1:**
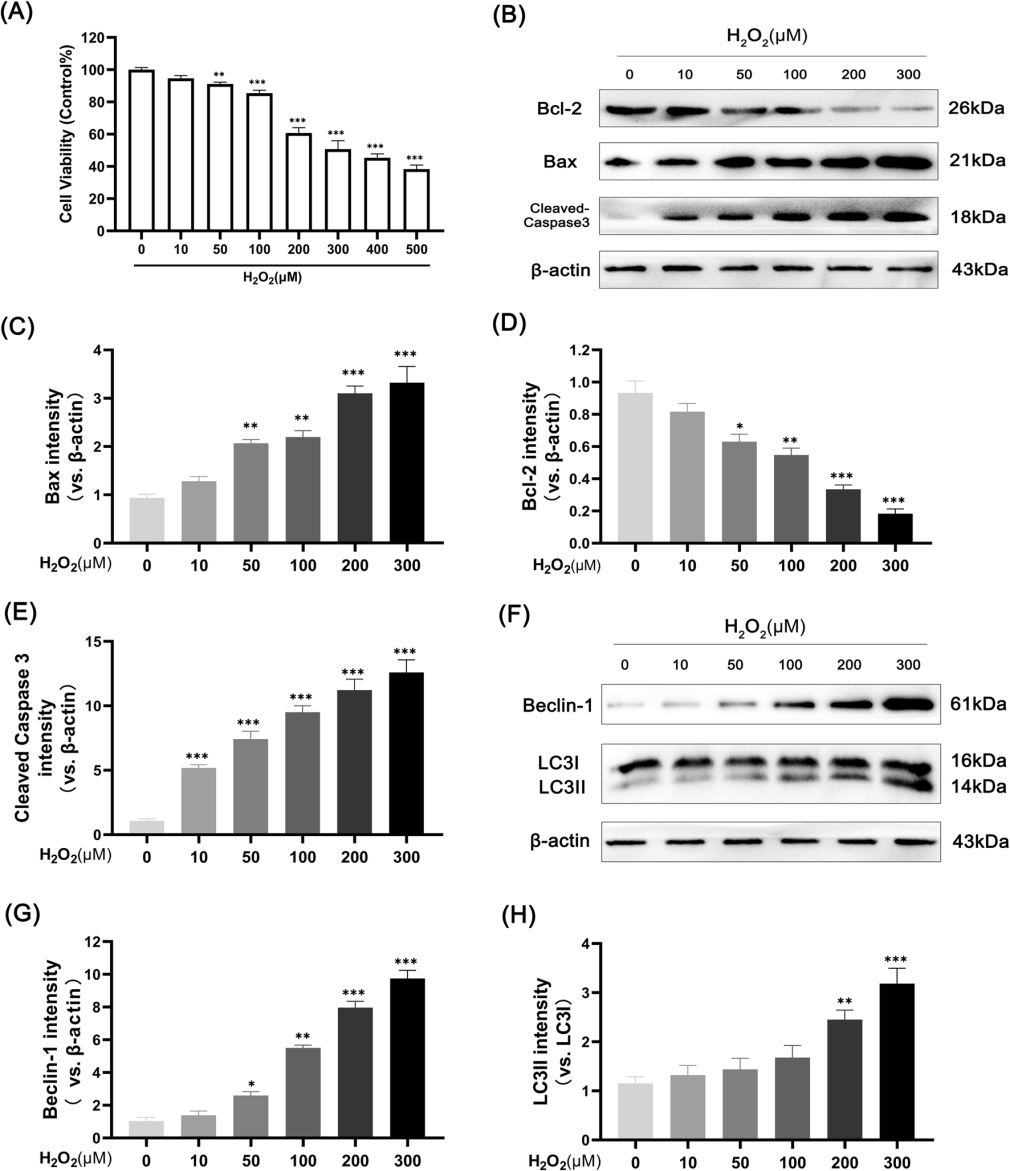
H_2_O_2_ induces apoptosis and activates autophagy in human chondrocytes. **A** Cell cytotoxicity detected by CCK-8 assay. Chondrocytes were treated with 0, 10, 50, 100, 200, 300, 400 and 500 μM H_2_O_2_ for 4 h. **B**–**E** western blot analysis and quantitative correlation analysis of Bax, cleaved caspase3 and Bcl-2 in chondrocytes. **F**–**H** western blot analysis and correlation quantitative analysis of LC3 and Beclin-1 in cells. Values are mean ± SD. *p < 0.05, **p < 0.01, and ***p < 0.001 versus the control group

The H_2_O_2_-induced chondrocyte damage model is considered a classical in vitro model of OA, which we made with reference to the experimental methods of previous studies [27, 28]. To assess the toxicity of H_2_O_2_ on chondrocytes, cells were stimulated with incremental doses of H_2_O_2_ for 4 h. The CCK-8 results indicated that chondrocytes were exposed to H_2_O_2_ for 4 h to inhibit activity in a dose-dependent fashion, and doses above 200 μM H_2_O_2_ had a significant inhibitory effect (Fig. 1A). Therefore, we selected 200 µM H_2_O_2_ for subsequent experiments. To search for the potential mechanism of reduced cell activity, we examined chondrocyte apoptosis and autophagy. To explore the apoptosis response of H_2_O_2_-stimulated human chondrocytes, we selected different doses of H_2_O_2_ (0, 10, 50, 100, 200 and 300 µM) to stimulate cells. After treatment for 24 h, the protein expression levels of Bax, cleaved-caspase3 and Bcl-2 were detected by western blot. As shown in Fig. 1B–E, H_2_O_2_ increased the expression of the proapoptotic proteins Bax and cleaved caspase3 and decreased the expression of the antiapoptotic protein Bcl-2 in a concentration-dependent manner. We also examined the effect of H_2_O_2_ on chondrocyte autophagy. The results showed that H_2_O_2_ enhanced the expression of the autophagy markers LC3-II/LC3-I and Beclin-1 in a concentration-dependent manner (Fig. 1F–H). The apoptosis and autophagy reactions of chondrocytes showed the same enhancement trend after H_2_O_2_ treatment. We speculate that overactivated autophagy exacerbated the apoptosis of chondrocytes.

### Effect of DAS on human chondrocyte viability and maintenance of cell phenotype

**Fig. 2:**
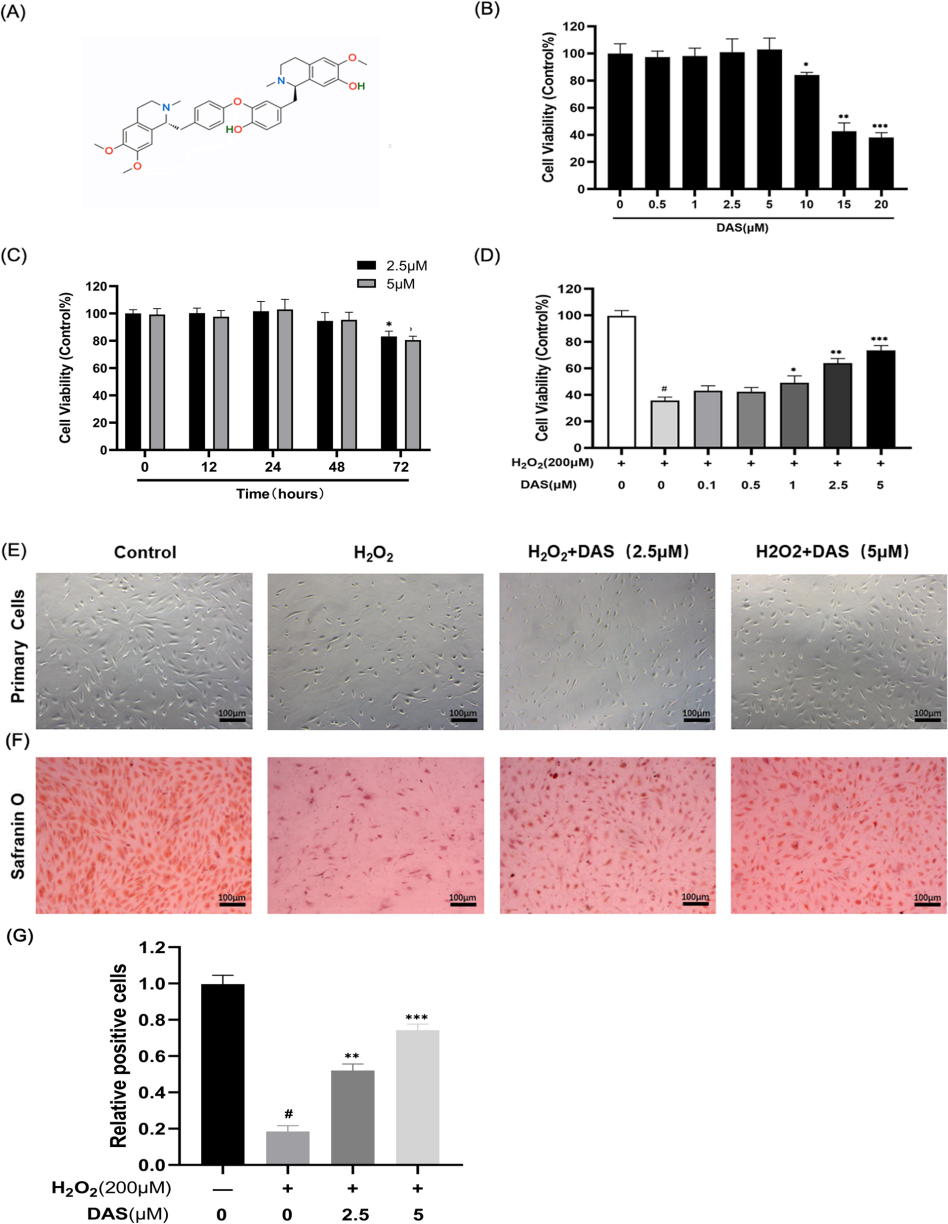
Protective effects of DAS on chondrocytes induced by H_2_O_2_. **A** The chemical structures of DAS. **B** Effects of DAS on chondrocyte activity. After being cultured with DAS (0.5, 1, 2.5, 5, 10, 15 and 20 μM) for 24 h, cell proliferation was determined by CCK8 assay. **C** Effects of DAS on chondrocyte activity. After being cultured with DAS (2.5 and 5 μM) for different treatment times (12, 24, 48 and 72 h), cell proliferation was determined by CCK8 assay. **D** Chondrocytes were pretreated with DAS (0.5, 1, 2.5, and 5 μM) for 24 h in the presence and absence of H_2_O_2_ (200 μM), and cell activity was measured by CCK8 assay. The values are mean ± SD. #p < 0.05 versus control group, *p < 0.05, **p < 0.01, and ***p < 0.001 versus control group. **E** Morphological analysis of chondrocytes after different treatments (magnification ×100, scale bar = 100 μm). **F** Chondrocyte phenotype, glycosaminoglycan production and matrix degradation were evaluated by Safranin O staining (magnification ×100, scale bar = 100 μm). **G** Statistical analysis of Safranin O staining

The chemical properties of DAS are shown in Fig. 2A. To assess the effect of DAS on chondrocytes, cells were treated with various doses of DAS for 24 h. According to Fig. 2B, DAS showed no significant toxicity on cells at doses of 0.5, 1, 2.5 and 5 μM. We further investigated the influence of two experimental concentrations of DAS (2.5 and 5 μM) on chondrocyte viability at different treatment times (12, 24, 48 and 72 h). As shown in Fig. 2C, DAS with a treatment time ≤ 48 h had no obvious toxic effect on chondrocytes, but when the treatment time was longer than 48 h, the cell activity was significantly reduced. When chondrocytes were pretreated with DAS at different concentrations (0.5, 1, 2.5 and 5 μM) for 24 h and then cultivated with or without H_2_O_2_ (200 μM) for 4 h, cell viability was significantly restored by the pretreatment, and the most protective concentration was 5 μM (Fig. 2D). Based on these data, DAS was added at concentrations of 2.5 and 5 μM was for further experiments. Next, morphological analysis of chondrocytes after different treatments was performed by phase-contrast microscopy. According to Fig. 2E, the number of cell colonies decreased after H_2_O_2_ treatment compared to the control value, while DAS effectively prevented H_2_O_2_-induced cell depletion. The effect of DAS on the chondrocyte phenotype was also tested by Safranin O staining, which yielded similar results. The application of DAS increased the secretion of glycosaminoglycan (GAG) by chondrocytes and prevented matrix degradation (Fig. 2F–G).

### DAS alleviates H_2_O_2_-induced human chondrocyte apoptosis

**Fig. 3:**
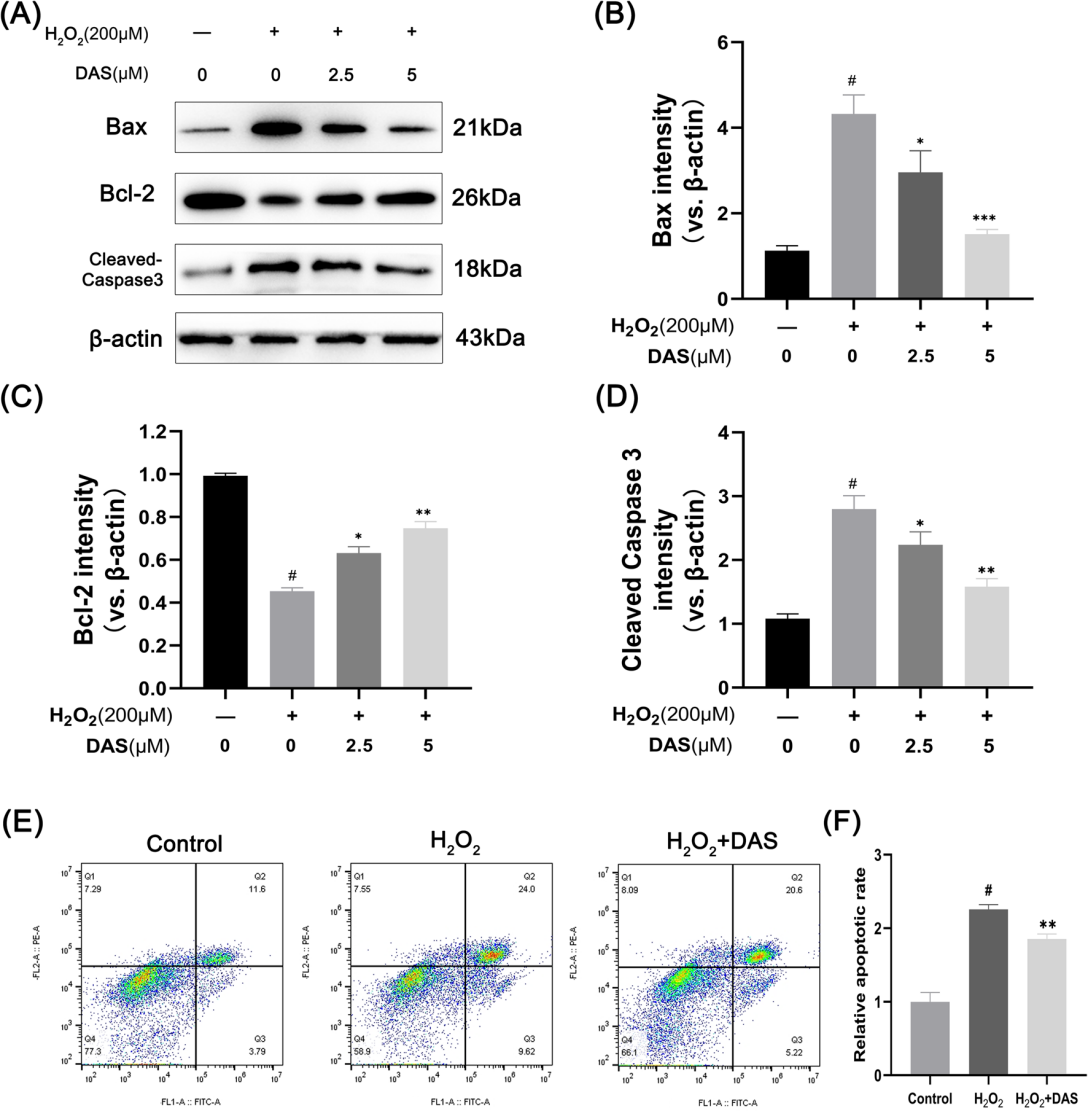
Daurisoline (DAS) protects chondrocytes from H_2_O_2_-induced apoptosis. **A**–**D** Western blot was performed to quantitatively analyze the expression of Bax, Bcl-2 and cleaved caspase-3. The values are mean ± SD. #p < 0.05 versus the control group. *p < 0.05, **p < 0.01, and ***p < 0.001 versus the control group. **E** Apoptosis was quantified by flow cytometry. (F) Relevant quantitative analysis of flow cytometry

To assess the effect of DAS on human chondrocyte apoptosis, we measured the expression of apoptosis-related factors by western blot. Chondrocytes were pretreated with DAS for 24 h and then incubated with or without H_2_O_2_ (200 μM) for 4 h. As shown in Fig. 3A–D, DAS (2.5 or 5 μM) inhibited the H_2_O_2_-induced upregulation of pro-apoptotic Bax and cleaved-caspase3 proteins, whereas it increased the expression of anti-apoptotic Bcl-2. Notably, treatment with 5 μM DAS resulted in a more marked recovery of apoptosis-related protein expression than the dose of 2.5 μM. Subsequently, flow cytometry was used to measure the proportion of apoptotic cells, and similar results were obtained. According to Fig. 3E–F, the apoptosis rate in the H_2_O_2_ group (33.62%) was significantly greater than that in the control group (15.39%), but the apoptosis rate decreased to 25.82% after DAS treatment. The above results showed that DAS protected chondrocytes from H_2_O_2_-induced apoptosis.

### DAS downregulates the level of autophagy in chondrocytes

**Fig. 4:**
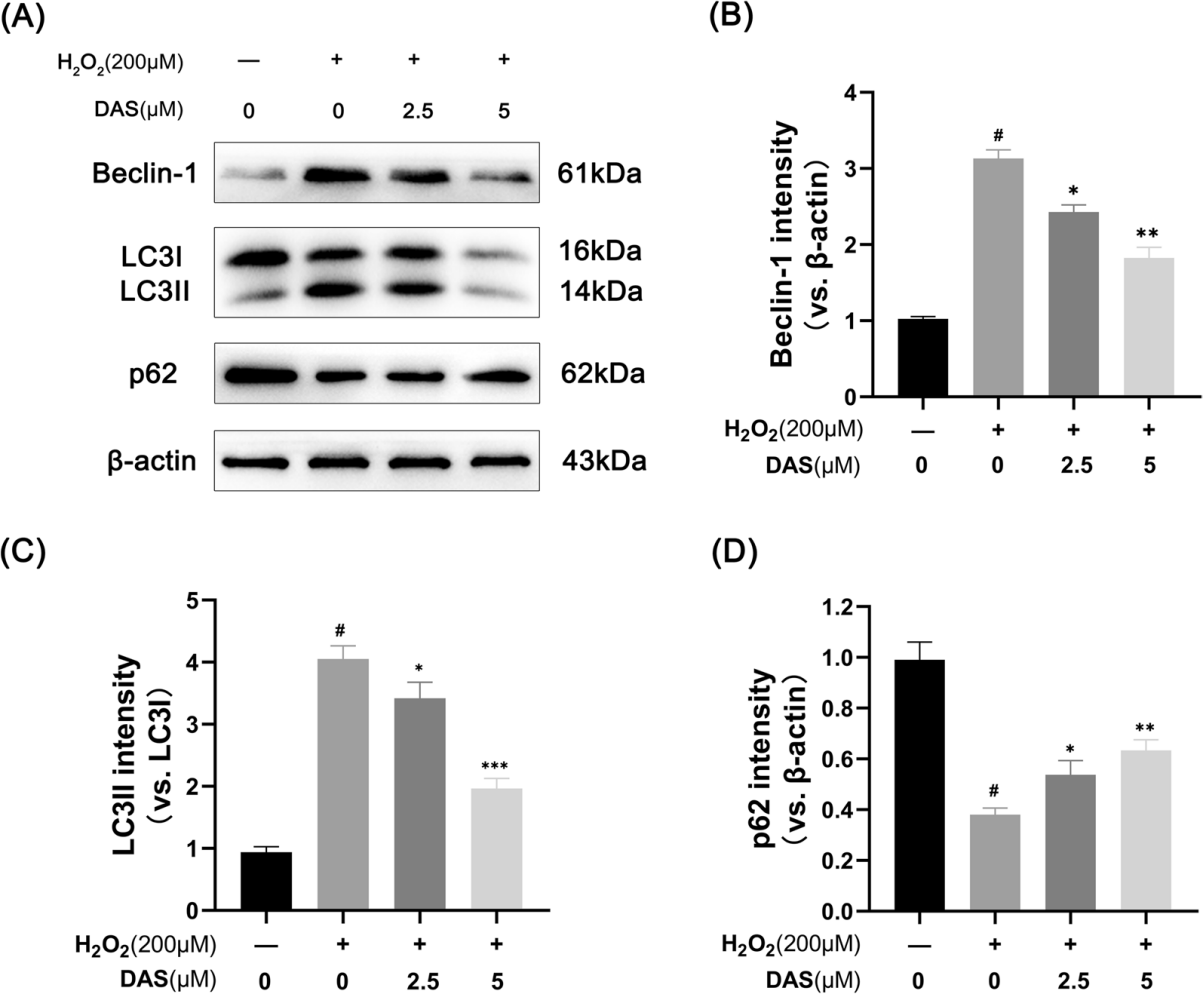
DAS inhibits H_2_O_2_-induced chondrocyte autophagy. **A**–**D** Western blot and correlation quantitative analysis of Beclin-1, LC3 and p62 in chondrocytes. The values are mean ± SD. #p < 0.05 versus the control group. *p < 0.05, **p < 0.01, and ***p < 0.001 versus the control group

**Fig. 5:**
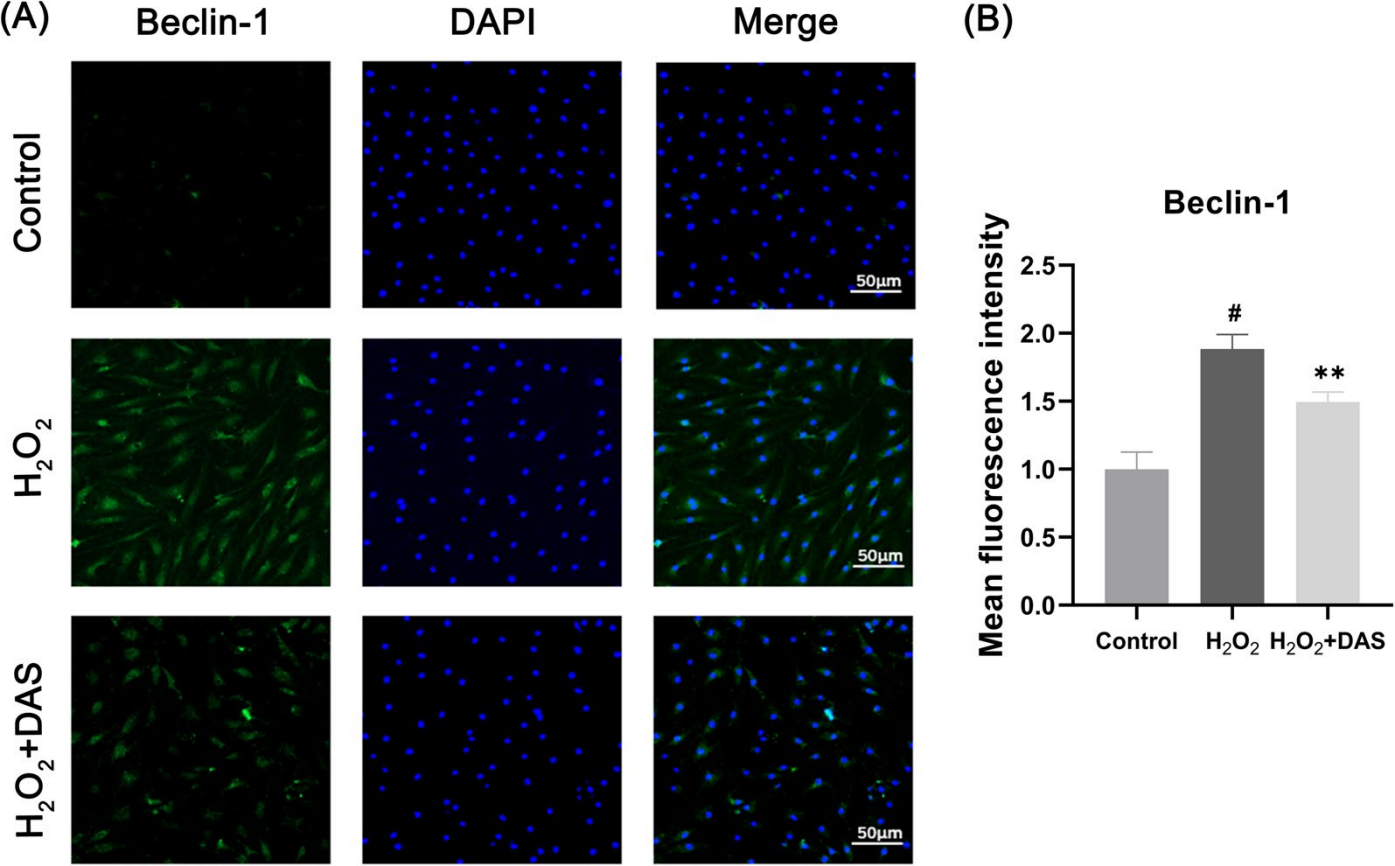
Beclin-1 expression as assayed by immunofluorescence. **A** Beclin-1 expression in chondrocytes was determined by immunofluorescence. Blue, DAPI; green, Beclin-1. Magnification ×400. Scale bar, 50 μm. **B** Fluorescence intensity quantification of Beclin1 expression

To clarify whether autophagy participates in the protective effect of DAS against H_2_O_2_-induced chondrocyte apoptosis, we detected the expression of autophagy markers. As shown in Fig. 4A–D, under H_2_O_2_ treatment, the autophagy markers LC3 and Beclin-1 increased, and the protein level of p62 was significantly reduced. Under DAS pretreatment (2.5 and 5 μM), the expression of LC3-II/LC3-I and Beclin-1 were significantly downregulated, while the p62 protein level was upregulated. To further confirm the role of DAS in chondrocyte autophagy, immunofluorescence was performed to detect the expression of Beclin-1. As shown in Fig. 5A, B, H2O2 (200 μM, 4 h) significantly upregulated the expression of Beclin-1 compared to that in the control group, while treatment with DAS (5 μM, 24 h) significantly antagonized the increase in H_2_O_2_-induced Beclin-1 expression. These results indicated that DAS inhibited H_2_O_2_-induced excessive autophagy.

### DAS activates the PI3K/AKT/mTOR signaling pathway

**Fig. 6:**
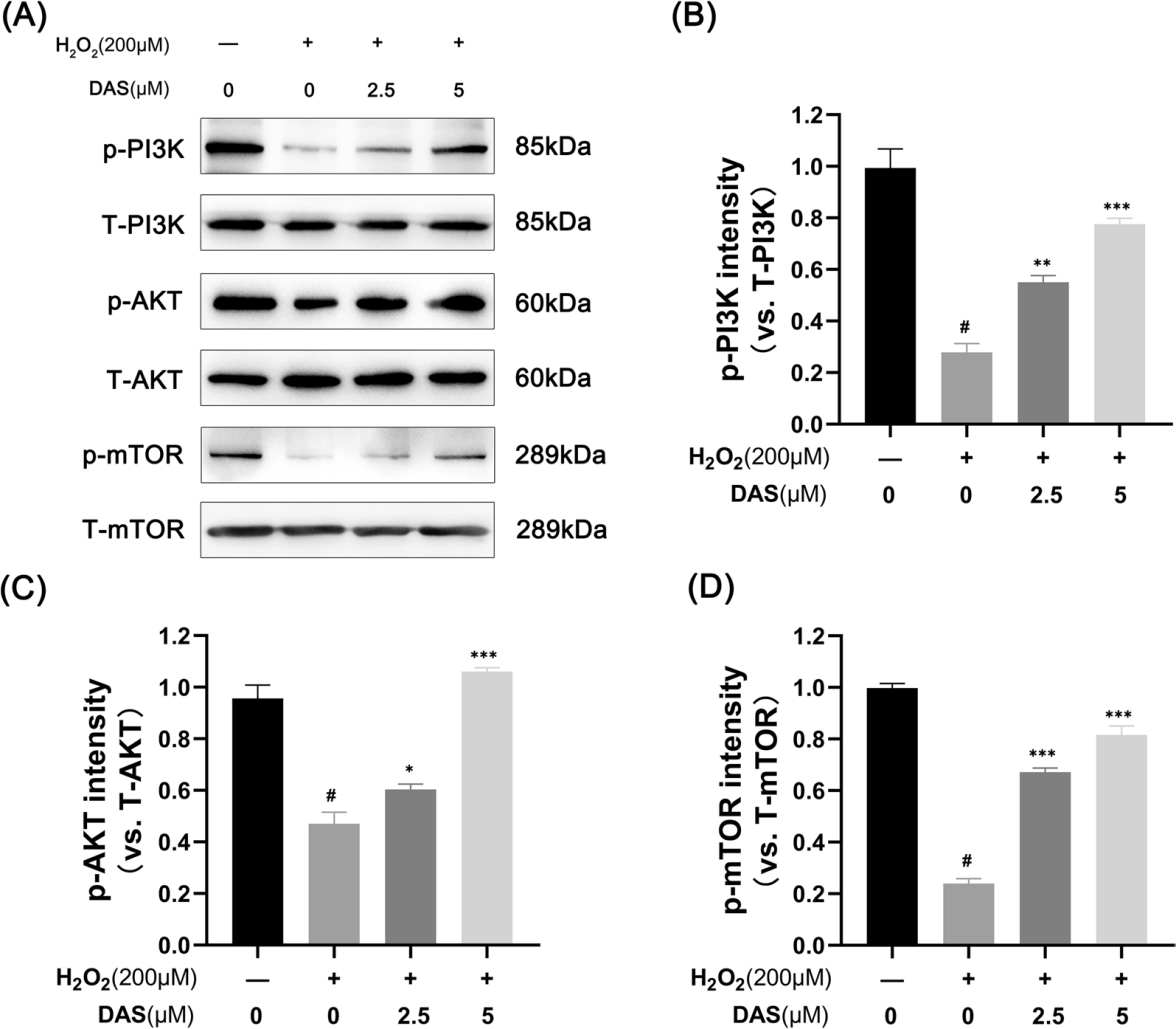
DAS activates the PI3K/AKT/mTOR signaling pathway. **A** The p-AKT, T-AKT, p-PI3K, T-PI3K, p-mTOR and T-mTOR expression levels in H_2_O_2_-stimulated chondrocytes with or without DAS were assayed by western blot. **B**–**D** Relevant quantitative analysis of the blots shown in **A**. The data represent the mean ± SD. #p < 0.05 versus the control group. *p < 0.05, **p < 0.01, and ***p < 0.001 versus the control group

The PI3K/AKT/mTOR signaling pathway has been reported to play a crucial role in chondrocyte apoptosis and autophagy. Therefore, to better understand the mechanism through which DAS prevents H_2_O_2_-induced chondrocyte apoptosis and autophagy damage, we investigated the involvement of the PI3K/AKT/mTOR signaling pathway by western blot. As shown in Fig. 6A–D, compared to the control group, p-PI3K, p-AKT and p-mTOR levels were reduced in chondrocytes treated with H_2_O_2_, while their expression was upregulated in chondrocytes treated with DAS (2.5 and 5 μM). These data suggest that the activation of PI3K/Akt/mTOR signaling may be related to the mechanism by which DAS inhibits autophagy injury.

### DAS inhibits autophagy marker expression through the PI3K/AKT/mTOR pathway and protects chondrocytes from apoptosis and matrix degradation

**Fig. 7:**
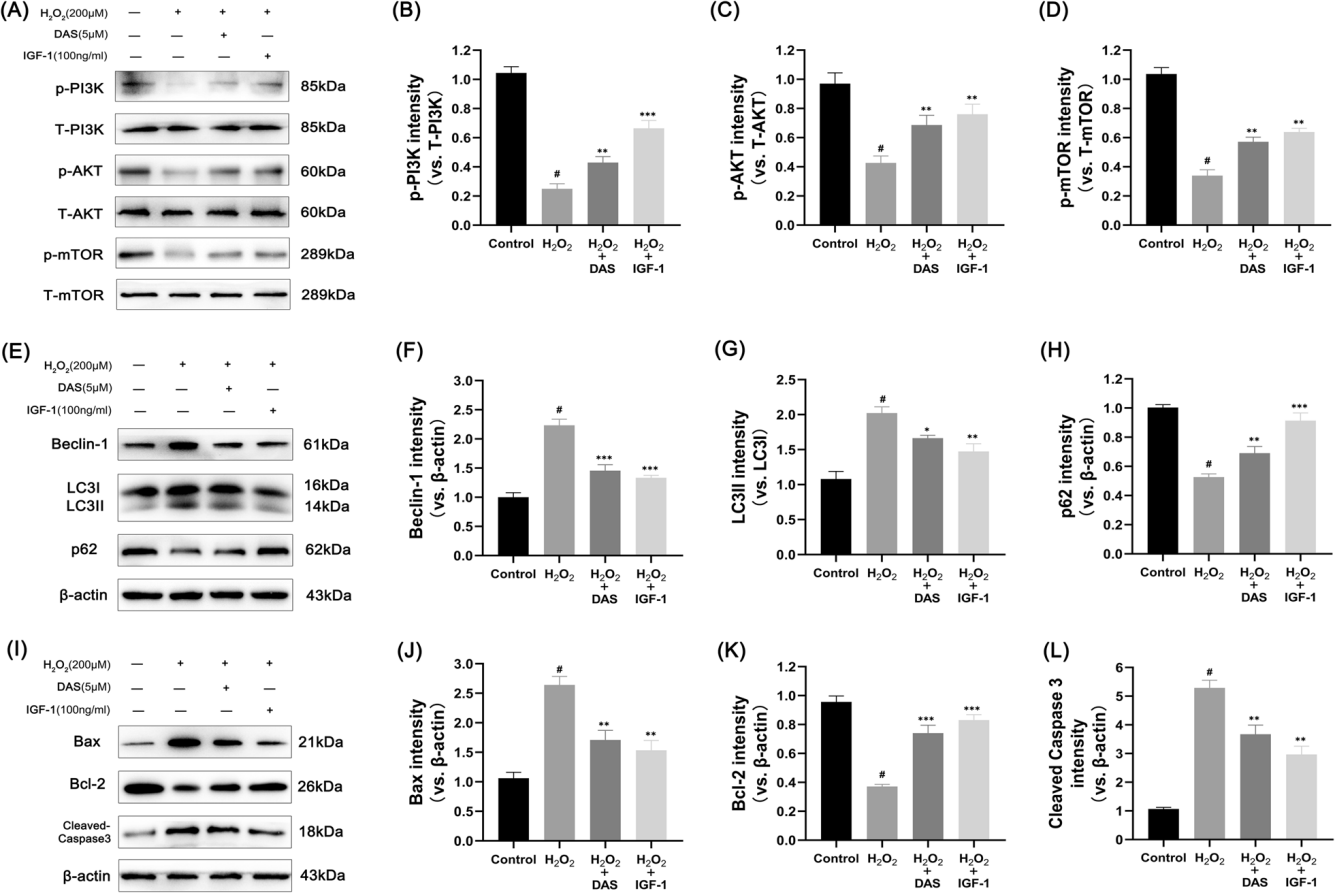
DAS inhibits autophagy markers and apoptosis-related factors through the PI3K/AKT/mTOR signaling pathway. **A**–**D** Western blot analysis of the protein levels of p-AKT, T-AKT, p-PI3K, T-PI3K, p-mTOR and T-mTOR and the quantification of associated proteins in the blots shown. **E**–**H** western blot and quantitative correlation analysis of Beclin-1, LC3 and p62 in chondrocytes. **I**–**L** Western blot was performed to quantitatively analyze the expression of Bax, Bcl-2 and cleaved caspase-3. The values represent the mean ± SD. #p < 0.05 versus the control group. *p < 0.05, **p < 0.01, and ***p < 0.001 versus the control group

To further verify whether DAS inhibited anomalous chondrocyte autophagy via the PI3K/AKT/mTOR signaling pathway, we treated chondrocytes with insulin-like growth 1 as a positive control (IGF-1, a PI3K activator). The cells were divided into the following 4 groups: control, H_2_O_2_, H_2_O_2_ + DAS and H_2_O_2_ + IGF-1. Then, the expression of relevant proteins was analyzed by western blot. As shown in Fig. 7A–D, H2O2 significantly inhibited phosphorylation of the PI3K/AKT/mTOR signaling pathway, while the separate addition of DAS and IGF-1 upregulated the phosphorylation levels of H_2_O_2_-inhibited PI3K, AKT, and mTOR to varying degrees. The results of autophagy marker expression showed that DAS and IGF-1 blocked autophagy. Western blot analysis showed that DAS and IGF-1 inhibited the autophagy-related proteins LC3 and Beclin-1 and promoted the expression of p62 (Fig. 7E–H). In addition, we found that DAS and IGF-1 reduced the stimulating effect of H_2_O_2_ on the expression of cleaved caspase-3 and Bax protein and enhanced the expression of the anti-apoptotic factor Bcl-2, as shown in Fig. 7I–L.

**Fig. 8:**
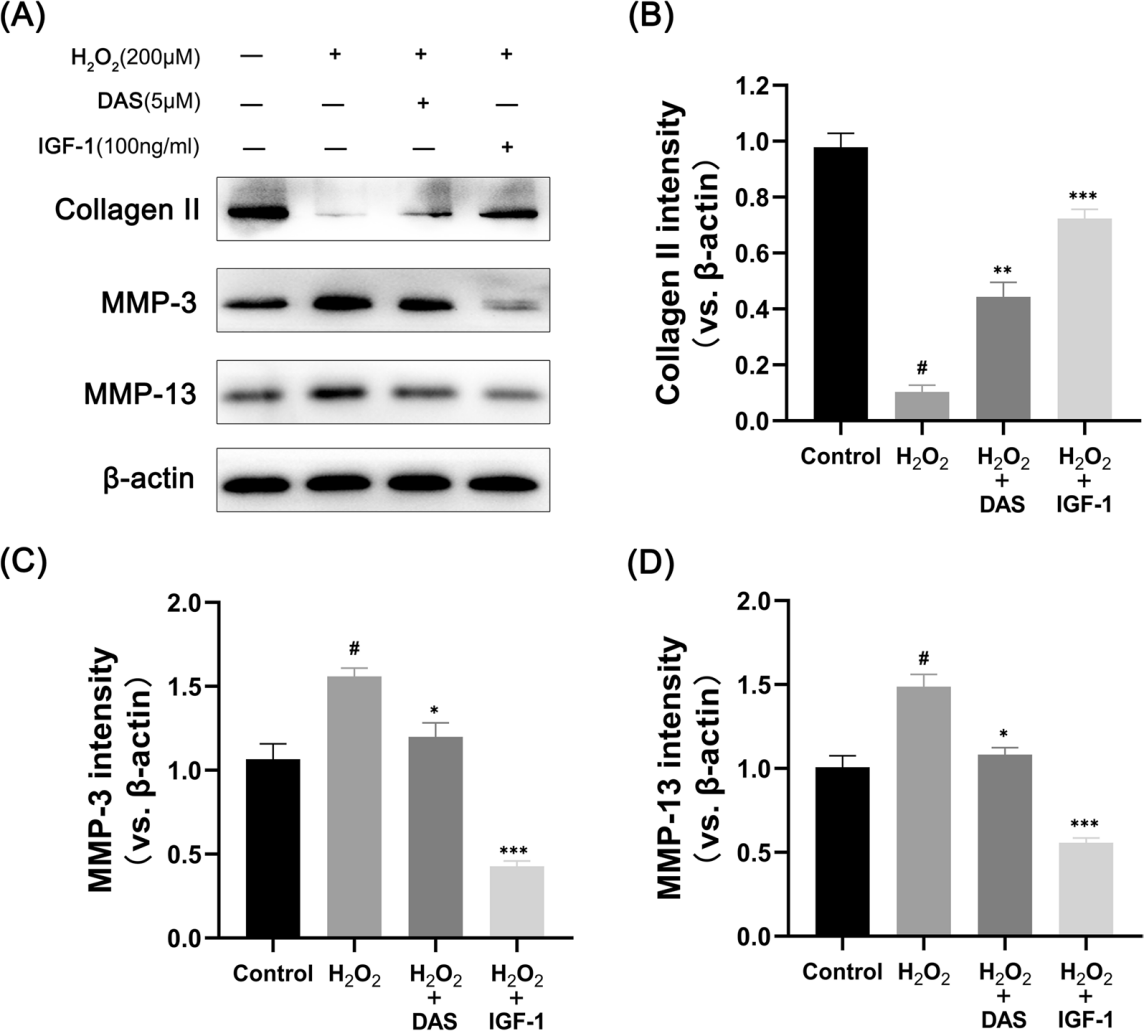
Effects of DAS on H_2_O_2_-induced matrix degradation of human chondrocytes. **A**–**D** Western blot and quantitative correlation analysis of collagen II, MMP-3 and MMP-13 in chondrocytes. The values represent the mean ± SD. #p < 0.05 versus the control group. *p < 0.05, **p < 0.01, and ***p < 0.001 versus the control group

As shown in Fig. 2E, we have preliminarily demonstrated the role of DAS in cartilage matrix metabolism. To further explore the effect of DAS on human chondrocytes, western blots were performed to detect the expression of the matrix component protein (collagen II) and matrix-degrading indicators (MMP-3 and MMP-13). The results showed that DAS significantly reversed the H_2_O_2_-induced downregulation of collagen II, inhibited the upregulation of MMP3 and MMP13, and blocked cartilage stromal decomposition (Fig. 8A–D). Therefore, DAS could protect human chondrocytes from stromal decomposition by restoring the H_2_O_2_-induced deletion of matrix component proteins and downregulating the expression of matrix-degrading proteins. In summary, DAS has a similar effect as IGF-1 and may block excessive autophagy and apoptosis of human chondrocytes by upregulating the activation of PI3K/AKT/mTOR signaling and alleviating cartilage matrix degradation.

## Discussion

Osteoarthritis is a chronic degenerative disease characterized by chronic intra-articular inflammation and articular cartilage degeneration, often resulting in chronic pain and joint disability, and is more prevalent in the elderly population [6, 29, 30]. While drugs such as nonsteroidal anti-inflammatory drugs (NSAIDs) can relieve chronic pain and swelling caused by OA, the deterioration of OA is not effectively reversed. In addition, the long-term application of such drugs can cause a range of side effects [31]. Therefore, finding safe and effective drugs to treat OA is imperative. In recent years, traditional Chinese medicine (TCM) has been recognized worldwide for its natural advantages [32]. Daurisoline is an isoquinoline alkaloid mainly extracted from the Chinese herbal medicine Rhizoma Menispermi, which exhibits a wide range of pharmacological effects in the treatment of cardiovascular and cerebrovascular diseases, including anti-inflammatory and antitumor effects [20, 22, 23]. In this study, we investigated the effect and molecular mechanisms of DAS in H_2_O_2_-induced oxidative stress injury of chondrocytes and found that DAS alleviated H_2_O_2_-induced autophagy, apoptosis and cartilage matrix degradation in chondrocytes. The potential mechanism was associated with the activation of the PI3K/AKT/mTOR pathway in human OA chondrocytes.

Oxidative stress is one of the leading causes of OA pathogenesis. It plays a major pathological driving role in ECM degradation, chondrocyte apoptosis and the inflammatory response [33–35]. It affects chondrocyte homeostasis through multiple pathways, such as mitochondrial dysfunction, telomerase shortening and DNA damage. In this study, we used H_2_O_2_ to induce oxidative stress injury in human OA chondrocytes to mimic the OA cell model. As expected, H_2_O_2_ markedly reduced chondrocyte viability and GAG secretion and significantly activated cell apoptosis. Moreover, the administration of DAS promoted the recovery of cell viability and phenotype and alleviated H_2_O_2_-induced chondrocyte apoptosis. These data indicate that DAS has a protective effect against H_2_O_2_-induced chondrocyte injury.

Autophagy is a critical biological process of self-digestion. It is believed to play a dual role in the pathogenesis of OA [36]. Many studies have shown that autophagy is intimately involved in the protection of cartilage lesions, and restoration of autophagy alleviates chondrocyte apoptosis in OA [37, 38]. On the other hand, when the degradation capacity of lysosomes is insufficient to eliminate the autophagosome contents, abnormal autophagy promotes the activation of the apoptotic cascade, and autophagy damage induces aging-related cell death and aggravates the progression of OA in patients [39, 40]. Therefore, autophagy in cartilage appears to be subtly regulated, and whether it is beneficial or harmful to the progression of OA depends on the balance between the amount of substrate and the capacity of the autophagy mechanism, and maintaining an appropriate level of autophagy under stress is crucial. In this study, we found that H_2_O_2_-induced autophagy markers LC3 and Beclin-1 were significantly increased in human chondrocytes, accompanied by increased apoptotic signals, such as increased Bax and decreased Bcl-2, suggesting that cartilage injury was caused by excessive autophagy-induced apoptosis. By detecting the protein expression of LC3II/LC3I, Beclin-1 and p62, we found that DAS strongly inhibited autophagy. In addition, immunofluorescence was used to detect Beclin-1 to determine whether DAS can inhibit chondrocyte autophagy. When autophagy was blocked by DAS, the expression of apoptosis-related proteins (Bax and cleaved caspase 3) was downregulated, the level of anti-apoptotic Bcl-2 was increased, and the percentage of apoptotic cells was also decreased. These findings suggest that DAS mitigated oxidative stress-induced apoptosis signaling by inhibiting excessive autophagy in chondrocytes, making this process a potential therapeutic target for OA.

Autophagy is controlled by the major negative regulator mTOR and its upstream regulator PI3K/Akt [41]. In addition to mediating autophagy, the PI3K/Akt/mTOR signaling pathway affects various physiological and pathological processes, such as cell proliferation, differentiation, inflammation, apoptosis, and cancer [42]. As the PI3K/Akt/mTOR pathway has been studied more, results have confirmed that the PI3K/AKT/mTOR pathway plays a role in apoptosis and autophagy of chondrocytes [43]. Considering the above reasons, we speculated that the regulatory effect of DAS on autophagy in osteoarthritis chondrocytes might be realized through the PI3K/AKT/mTOR signaling pathway. In our study, DAS significantly activated H_2_O_2_-induced inhibition of the PI3K/AKT/mTOR signaling pathway. Furthermore, IGF-1 was employed to activate the PI3K/Akt/mTOR signaling pathway as a positive control. Our results suggested that both DAS and IGF-1 treatment reversed H_2_O_2_-induced autophagy, apoptosis and cartilage matrix degradation. Therefore, these data indicate that DAS can reverse H_2_O_2_-induced adverse outcomes through the PI3K/Akt/mTOR signaling pathway, establishing the relationship between DAS and the PI3K/Akt/mTOR signaling pathway. Activation of mTOR is known to inhibit autophagosome formation, and inhibition of mTOR pathway signaling leads to cell death related to apoptosis and autophagy [44, 45]. Therefore, we suspect that the inhibition of apoptosis induced by excessive autophagy is synergistic with the antiapoptotic effect of PI3K/AKT/mTOR signaling pathway activation, which makes the antiapoptotic effect of DAS more significant.

In summary, these results suggest that the potential mechanism by which DAS alleviates autophagy and apoptosis in chondrocytes is related to the activation of the PI3K/AKT/mTOR pathway. Whether PI3K/AKT/mTOR is a direct target of DAS remains unknown. Further studies are needed to elucidate the exact mechanism by which DAS regulates the PI3K/AKT/mTOR signaling pathway. In addition, the data obtained from in vitro experiments may differ from the results of in vivo experiments. Therefore, the curative effect of DAS on OA needs to be further studied.

## Conclusion

This study preliminarily demonstrated that DAS inhibits chondrocyte apoptosis and improves inflammatory responses and matrix degradation in vitro by inhibiting H_2_O_2_-induced excessive autophagy. These effects may be mediated by activation of the PI3K/AKT/mTOR pathway. Our findings indicate the great potential of DAS in the treatment of OA. Future studies should focus on the efficacy and more comprehensive mechanisms of DAS in vivo, to determine whether there is strong evidence supporting the application of DAS in clinical trials.

## Data Availability

Data are available on request from the authors.
