# Correction: Daurisoline attenuates H2O2-induced chondrocyte autophagy by activating the PI3 K/Akt/mTOR signaling pathway

**DOI:** 10.1186/s13018-025-05845-6

**Published:** 2025-05-16

**Authors:** Yang Zhang, Wenguang Liu, Zhonghao Liu, Yi Liu

**Affiliations:** Department of Orthopaedics, The Second Hospital, Cheeloo College of Medicine, Shandong University, 247 Beiyuan Street, Jinan, Shandong 250033 People’s Republic of China

**Correction: J Orthop Surg Res 18:248 (2023)**

**https://doi.org/10.1186/s13018-023–03717-5**

In this article Fig. 7 appeared incorrectly and has now been corrected in the original publication. For completeness and transparency, the old incorrect versions are displayed below.

Incorrect Fig. 7

**Fig. 7:**
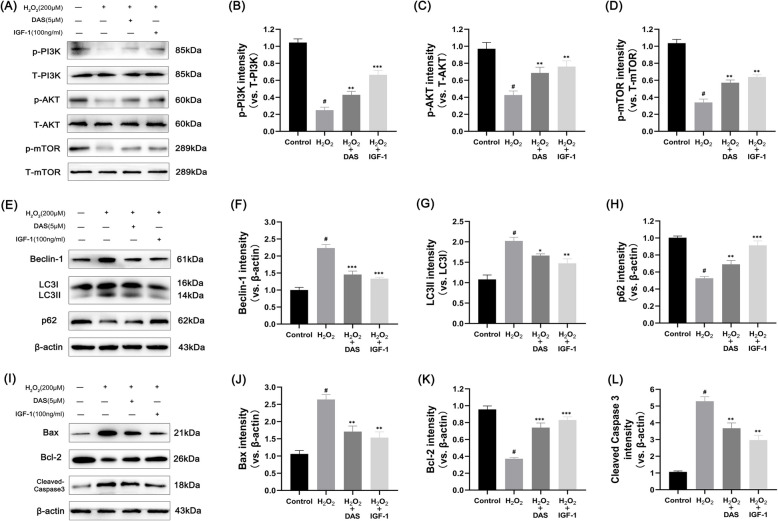
DAS inhibits autophagy markers and apoptosis-related factors through the PI3K/AKT/mTOR signaling pathway. **A**–**D** Western blot analysis of the protein levels of p-AKT, T-AKT, p-PI3K, T-PI3K, p-mTOR and T-mTOR and the quantification of associated proteins in the blots shown. **E**–**H** western blot and quantitative correlation analysis of Beclin-1, LC3 and p62 in chondrocytes. **I**–**L** Western blot was performed to quantitatively analyze the expression of Bax, Bcl-2 and cleaved caspase-3. The values represent the mean ± SD. #p < 0.05 versus the control group. *p < 0.05, **p < 0.01, and ***p < 0.001 versus the control group

Corrected Fig. 7

**Fig. 7:**
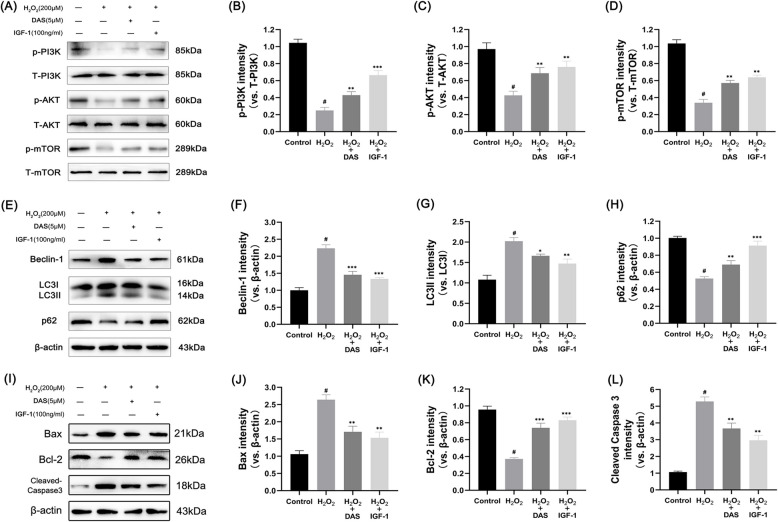
DAS inhibits autophagy markers and apoptosis-related factors through the PI3K/AKT/mTOR signaling pathway. **A**–**D** Western blot analysis of the protein levels of p-AKT, T-AKT, p-PI3K, T-PI3K, p-mTOR and T-mTOR and the quantification of associated proteins in the blots shown. **E**–**H** western blot and quantitative correlation analysis of Beclin-1, LC3 and p62 in chondrocytes. **I**–**L** Western blot was performed to quantitatively analyze the expression of Bax, Bcl-2 and cleaved caspase-3. The values represent the mean ± SD. #p < 0.05 versus the control group. *p < 0.05, **p < 0.01, and ***p < 0.001 versus the control group

The original article has been corrected.

